# Questioning the IGF1 receptor’s assigned role in CRC – a case for rehabilitation?

**DOI:** 10.1186/s12885-020-07173-w

**Published:** 2020-07-29

**Authors:** Steffen M. Heckl, Marie Pellinghaus, Hans-Michael Behrens, Sandra Krüger, Stefan Schreiber, Christoph Röcken

**Affiliations:** 1 Department of Internal Medicine I, University Hospital Schleswig-Holstein, Kiel, Germany; 2 Department of Pathology, Christian-Albrechts-University, Kiel, Germany

**Keywords:** Colorectal cancer, IGF1 receptor, Cancer risk factor, Cancer prognosis, Cancer therapy

## Abstract

**Background:**

The insulin-like growth factor 1 receptor (IGF1R) is suspected to be involved in colorectal carcinogenesis and has been associated with worse survival in colorectal cancer (CRC). We hypothesized that the alleged suspect might be in truth beyond any suspicion. We investigated if the expression of the IGF1R in CRC correlates with (1) clinicopathological patient characteristics, including survival, and hence is involved in colon cancer biology; (2) the expression of the IGF1R in CRC is linked to the expression of the insulin receptor (IR).

**Methods:**

We evaluated 4497 CRC samples from 1499 patients for the expression of IGF1R in tumor cells by immunohistochemistry. Cytoplasmic (cCC-IGF1R) and membranous (mCC-IGF1R) immunostaining was evaluated by employing a modified HistoScore (HScore), which was dichotomized into low or high IGF1R expressions. The IGF1R status was correlated with clinicopathological patient characteristics, survival and the IR expression status.

**Results:**

cCC-IGF1R and mCC-IGF1R (HScore> 0) were found in 85.4 and 60.8% of all CRCs. After dichotomization of the HScores, 54.9 and 48.6% were classified as cCC-IGF1R-high and mCC-IGF1R-high, respectively. IGF1R was associated with tumor localization, local tumor growth, lymphatic vessel invasion, grading, mismatch repair protein expression status and IR-expression. We found no significant association with overall or tumor-specific survival, with a tendency for an even improved overall survival for cCC-IGF1R.

**Conclusions:**

IGF1R expression is frequent and biologically relevant in CRC, but does not correlate with patient survival. The IGF1R might be beyond suspicion in CRC after all.

## Background

The insulin-like growth factor 1 receptor (IGF1R) is suspected to be involved in colorectal carcinogenesis and has been associated with worse survival in colorectal cancer (CRC) [1, 2].

The IGF1R is known to interact with the insulin receptor (IR), thereby constituting the IGF1R−/IR-axis [3]. We recently reported that IR expression is associated with distinct clinicopathological parameters and survival in CRC [4]. Surprisingly, CRC patients with IR expression in tumor cells proved to show longer overall and tumor-specific survival rates than those with IR negative tumors. We therefore wondered why the IGF1R – which shares common ligands and signal transduction pathways with the IR – should contribute to worse survival in CRC? We hypothesized that the alleged suspect might be in truth beyond any suspicion.

We decided to gather as much evidence as possible, knowing that previous studies about IGF1R expression in CRC had been based upon study cohorts of limited size (Nakamura et al. *n* = 116 CRC patients [5]; Takahari et al. *n* = 91 CRC patients [2]; Shiratsuchi et al. *n* = 210 CRC patients [6]). Using a study population of 1499 patients we aimed to investigate the effects of IGF1R expression in CRC more extensively. An extensive analysis of IGF1R expression in CRC might help to further unravel the reasons for the striking ineffectiveness of IGF1R-directed therapy in CRC clinical trials [7–9].

In this study we tested the following hypotheses: the expression of IGF1R in CRC correlates with (1) clinicopathological patient characteristics, including survival, and hence is involved in colon cancer biology; (2) the expression of IGF1R in CRC is linked to the expression of the IR.

## Methods

### Study population and histology

From the archive of the Institute of Pathology, University Hospital Schleswig-Holstein, Kiel, we retrieved all samples of patients who had undergone oncologic resections of primary CRCs between 1995 and 2011. All tissue samples had been obtained from routine therapeutic surgeries. After fixation in neutral buffered formalin, all tissue specimens had been embedded in paraffin. Paraffin sections were subsequently cut and then stained with hematoxylin and eosin (H&E). The World Health Organization criteria were employed for histological classification. Board certified pathologists classified the tumor-node-metastasis stage according to the criteria of the *union internationale contre le cancer* (UICC; 7th edition) [10]. After study inclusion, all patient data were pseudonymized. Patients were excluded (1) if syn- or metachronous colon cancer was documented and (2) if the sample did not contain tumor cells.

Tissue microarray construction.

Tissue microarrays (TMA) were constructed from formalin-fixed and paraffin-embedded tissue samples as previously described [11]. H&E-stained tissue slides of each CRC sample were examined and three separate representative areas were selected randomly from the tumor area of the donor paraffin block(s) by a board-certified pathologist. A core was punched and transferred to the recipient paraffin block, thereby yielding three representative tissue cores per CRC patient within our TMAs. Successful transfer of tumor tissue was verified by H&E-staining of serial sections obtained from the TMAs.

Immunohistochemistry.

**Fig. 1:**
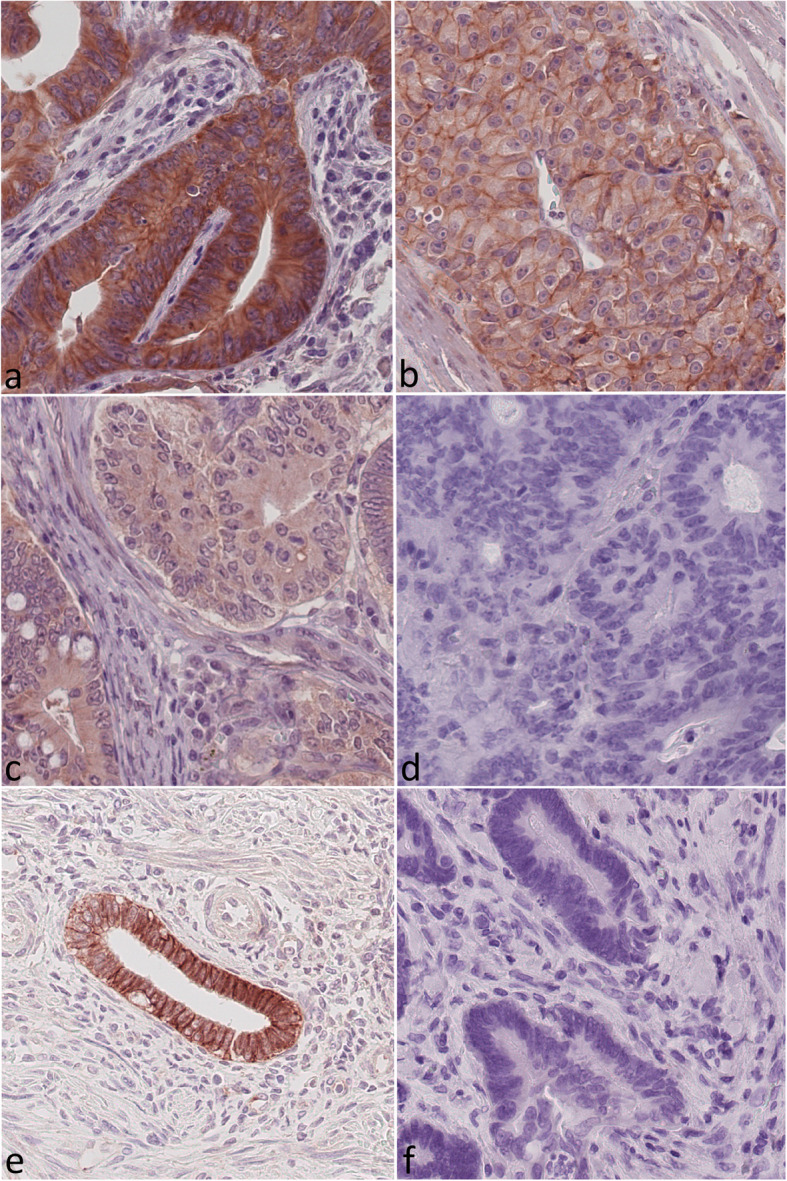
Insulin-like growth factor 1 receptor (IGF1R) immunoreactivity. Colorectal carcinoma samples showing (**a**) strong cytoplasmic (2+) and strong membranous (2+) staining, (**b**) weak (1+) cytoplasmic and weak (1+) membranous staining, (**c**) weak cytoplasmic (1+) and no (0) membranous staining, and (**d**) neither cytoplasmic nor membranous insulin-like growth factor 1 receptor (IGF1R) staining. IGF1R expression in endometrial cells (proliferative phase) served as a positive control (**e**) and the omission of the primary antibody served as the negative control (**f**). Original magnification **a**-**d**: 400x

Paraffin sections were deparaffinized and boiled in EDTA buffer (pH 9.0) for 1 min at 125 °C. All tissue slides were washed with Tris-buffered saline (TBS) and then blocked with hydrogen peroxide block (Thermo Fisher Scientific) for 15 min. After washing with TBS and subsequent incubation with Ultra V Block (Thermo Fisher Scientific) for 5 min, all slides were incubated with the primary antibody. The incubation with the primary antibody was performed for 30 min at room temperature, followed by an incubation overnight at 4 °C. The IGF1-receptor β antibody (rabbit monoclonal; clone D406W; Cell Signaling Technologies, Danvers, USA) was used with a 1:50 dilution. The ImmPRESS reagent peroxidase universal anti-mouse/rabbit Ig-MP-7500 (Vector Laboratories, Burlingame CA, USA) served as the peroxidase conjugated secondary antibody. The ImmPact NovaRed peroxidase substrate SK-4805 Kit (Vector Laboratories, Burlingame CA, USA) was used for the visualization of immunoreactions. All tissue slides were counterstained with hematoxylin. Negative controls were generated by omission of the primary antibody (Fig. 1). Endometrium samples served as positive controls (Fig. 1).

### Evaluation of IGF1 receptor immunostaining

At first, the entire series of 4497 TMA spots was screened to assess minimum and maximum staining intensities achieved with the staining protocol. Finally, a three-tired (0, 1+, 2+) scoring system of the staining intensity was considered to be appropriate and samples representing each staining intensity were selected as references for further assessment (Fig. 1). The evaluation distinguished between cytoplasmic (cCC-IGF1R) and membranous immunostaining (mCC-IGF1R) of the tumor cells. Subsequently, the whole study population was evaluated in depth. The three cores of each CRC specimen were treated as a single case. Subsequently, a modified HistoScore (HScore) was employed for the evaluation of IGF1R immunostaining. First the intensity of cytoplasmic and membranous IGF1R immunostaining, respectively, within tumor cells was evaluated and categorized as absent 0 (=no evidence of staining), weak (1+) and strong (2+). Secondly, the percentage of tumor cells with no (0), weak (1+), or strong (2+) immunostaining within each given tumor sample was estimated. The percentage of immunostained cells always added up to 100% according to the following formula: % (0) + % (1+) + % (2+) = 100% tumor cells. Subsequently, an HScore was calculated using the following formula: HScore = [0 x percentage of immunonegative tumor cells] + [1 x percentage of weakly stained tumor cells] + [2 x percentage of strongly stained tumor cells]. The maximum possible HScore was 200, if all tumor cells within a sample showed a strong (2+) immunostaining. The HScore served to improve the stratification of our samples, by separating more distinctively samples of low and of high immunostaining intensities. Finally, the cohort was split at the median HScore in high or low IGF1R expression. One person scored all samples (MP) and repeatedly compared the scoring with the study’s predetermined reference samples (Fig. 1) in order to decrease intra-observer variability. In the case of ambiguous immunostaining results, a second investigator (CR/SH) from the team was referred to and a consensus was reached. The scoring was reviewed on a random sample basis by a second investigator (CR/SH) in order to validate the consistency of the evaluation process.

### Assessment of the insulin receptor status

The IR status was assessed as previously described [4]. In brief, a monoclonal anti-insulin-receptor antibody (rabbit, clone 4B8; Cell Signaling Technologies, Danvers, USA; dilution 1:50; manual immunostaining) was used for immunohistochemistry. IR expression in vessels and in tumor cells was evaluated. With respect to IR expression in tumor cells, immunostaining was classified as either being negative, if no staining was evident, or positive, if any immunostaining was present. IR expression in cancer vasculature was scored ranging from absent (0) to strong (3+). Vascular IR expression was categorized into absent (0), weak (1+), moderate (2+) and strong (3+). The IR expression data as such has been published elsewhere [4] and has now been correlated with the new IGF1R expression data of the present study. The comparative analysis of IGF1R- and IR-expression was based on the same TMA cores.

### Assessment of DNA mismatch repair protein immunostaining

The expression of DNA mismatch repair proteins (MMR) MLH1, PMS2, MSH6 and MSH2 were assessed according to the algorithm suggested by Remo et al. [12] as previously described [4]. The algorithm is based upon the evaluation of nuclear staining within tumor cells. MMR deficient (dMMR) and MMR proficient CRCs were discriminated. Occasional cases of inconclusive MMR staining results were excluded, e.g. due to the absence of a positive internal control, or due to technical artifacts.

### *KRAS* genotyping

For genotyping one representative tissue section and the corresponding paraffin block were chosen from the resection specimens. The tumor area was marked on the H&E-stained slide. The percentage of tumor tissue in the marked area and the relative amounts of the histoanatomical components of the tumor, i.e. tumor cells and desmoplastic stroma were estimated visually to guarantee a valid tumor cell content. Genomic DNA was then extracted from formalin-fixed and paraffin-embedded tissue with the QIAamp DNA mini kit (Qiagen, Hilden, Germany) following the manufacturer’s instructions. To ensure a tumor cell percentage of > 40% in the analyzed specimens the tissue sections were manually microdissected prior to DNA extraction. For mutational analysis of codons 12 and 13 of the KRAS gene a 179 bp fragment was amplified by polymerase chain reaction (PCR) using the primers 5′-AGGCCTGCTGAAAATGACTGAATA-3′ (sense) and 5′- CTGTATCAAAGAATGGTCCT GCAC-3′ (antisense).15 PCR products were purified using the Nucleospin Extract II kit (Macherey-Nagel, Düren, Germany) and both strands sequenced by dye terminator cycle sequencing (BigDye Terminator v1.1 Cycle Sequencing kit, Applied Biosystems, Darmstadt, Germany) with the primers used for PCR amplification. The sequencing products were analyzed on an ABI Prism 310 Genetic Analyzer (Applied Biosystems). The results were confirmed by pyrosequencing on a PyroMark Q24 instrument as described by Ogino et al. [13]. Mutational analyses of codon 61 of the KRAS gene were done by pyrosequencing. In brief, specific DNA fragments of the individual genes were amplified by PCR using the primers 5′-AATTGATGGAGAAACCTGTCTCTT-3′ and 5′-TCCTCATGTACTGGTCCCTCATT-3′ (KRAS, codon 61). The resulting PCR products were sequenced on a PyroMark Q24 instrument with the sequencing primers 5′-GGATATTCTCGACACAGC-3′ (KRAS, codon 61), The KRAS-genotyping had been certified by an external quality assurance program done by the German Society of Pathology and the Bundesverband Deutscher Pathologen e.V. (www.dgp-berlin.de).

### Statistical analyses

For statistical analyses SPSS version 24.0 (IBM Corp., Armonk, NY, USA) was employed. Fisher’s exact test was used to test the correlation between non-ordinal clinicopathological patient characteristics and the mCC-IGF1R-status, or the cCC-IGF1R-status, respectively. Fisher’s exact test also served to test correlations between the IR status and the mCC-IGF1R-status, or the cCC-IGF1R-status. Variables of ordinal scale such as the T category, N category, UICC stage and tumor grading were tested with Kendall’s tau test. The Kaplan-Meier method was used to determine median survival with 95% intervals. The log-rank test was employed to test differences between median survivals. A *p*-value of ≤0.05 was defined to be significant. All *p* values are displayed without correction. We applied the Siemes (Benjamini-Hochberg) procedure to compensate a false discovery rate within the correlations. *P*-values having lost significance are marked.

## Results

### Study population

The clinicopathological characteristics of our patient collective are summarized in Table 1. 730 women and 769 men comprised the patient population with an overall median age of 71 years (range 26–95 years). During the follow-up 980 out of 1499 patients had died, with a median follow-up time of 58.7 months.

**Table 1:** Correlation between clinicopathological patient characteristics and the expression of insulin-like growth factor receptor 1 (IGF1R) in tumor cells. (**a**) Fisher’s exact test (b) Kendall’s tau test (c) Log-rank test. Abbreviations: n.c. = not calculated. * *p* values having lost significance according to the Siemes (Benjamini-Hochberg) procedure for multiple testing

		Total	Membranous IGF1R expression		Cytoplasmic IGF1R expression	
			lowHScore ≤ 10lowHScore ≤ 10lowHScore ≤ 10lowHScore ≤ 10	highHScore > 10highHScore > 10highHScore > 10highHScore > 10	lowHScore < 90lowHScore < 90lowHScore < 90lowHScore < 90	highHScore ≥ 90highHScore ≥ 90highHScore ≥ 90highHScore ≥ 90
		n(%)	n(%)	n(%)	n(%)	n(%)
**Gender**	n *p*-Value (a)	1499	1499	0.836	1499	0.350
Male		769 (51.3)	398 (51.8)	371 (48.2)	356 (46.3)	413 (53.7)
Female		730 (48.7)	373 (51.1)	357 (48.9)	320 (43.8)	410 (56.2)
**Age Group**	n *p*-Value (a)	1499	1499	0.877	1499	0.324
< 71 years		738 (49.2)	378 (51.2)	360 (48.8)	323 (43.8)	415 (56.2)
≥ 71 years		761 (50.8)	393 (51.6)	368 (48.4)	353 (46.4)	408 (53.6)
**Localization**	n *p*-Value (a)	1422	1422	**< 0.001**	1422	**< 0.001**
Left-sided		860 (60.5)	394 (45.8)	466 (54.2)	347 (40.3)	513 (59.7)
Right-sided		562 (39.5)	336 (59.8)	226 (40.2)	287 (51.1)	275 (48.9)
**T-Category**	n *p*-Value (b)	1499	1499	0.678	1499	**< 0.001**
T1		71 (4.7)	41 (57.7)	30 (42.3)	26 (36.6)	45 (63.4)
T2		316 (21.1)	158 (50.0)	158 (50.0)	115 (36.4)	201 (63.6)
T3		863 (57.6)	448 (51.9)	415 (48.1)	407 (47.2)	456 (52.8)
T4a/b		249 (16.6)	124 (49.8)	125 (50.2)	128 (51.4)	121 (48.6)
**T-Category (grouped)**	n *p*-Value (a)	1499	1499	1.000		**< 0.001**
T1/T2		387 (25.8)	199 (51.4)	188 (48.6)	141 (36.4)	246 (63.6)
T3/T4a/T4b		1112 (74.2)	572 (51.4)	540 (48.6)	535 (48.1)	577 (51.9)
**N-Category**	n *p*-Value (b)	1480	1480	0.197	1480	0.047*
N0		821 (55.5)	429 (52.3)	392 (47.7)	354 (43.1)	467 (56.9)
N1a/b/c		316 (21.3)	171 (54.1)	145 (45.9)	140 (44.3)	176 (55.7)
N2a/b		343 (23.2)	160 (46.6)	183 (53.4)	172 (50.1)	171 (49.9)
**N-Category**	n *p*-Value (a)	1480	1480	0.464	1480	0.115
N0		821 (55.5)	429 (52.3)	392 (47.7)	354 (43.1)	467 (56.9)
N+ (N1a/b/c, N2a/b)		659 (44.5)	331 (50.2)	328 (49.8)	312 (47.3)	347 (52.7)
**M-Category**	n *p*-Value (a)	1499	1499	0.331	1499	0.871
M1		172 (11.5)	82 (47.7)	90 (52.3)	79 (45.9)	93 (54.1)
MX		1327 (88.5)	689 (51.9)	638 (48.1)	597 (45.0)	730 (55.0)
**UICC Stage**	n *p*-Value (b)	1499	1499	0.358	1499	0.025*
I		310 (20.6)	161 (51.9)	149 (48.1)	116 (37.4)	194 (62.6)
IIA/B/C		494 (33.0)	261 (52.8)	233 (47.2)	231 (46.8)	263 (53.2)
IIIA/B/C		524 (35.0)	268 (51.1)	256 (48.9)	251 (47.9)	273 (52.1)
IVA/B		171 (11.4)	81 (47.4)	90 (52.6)	78 (45.6)	93 (54.4)
**L-Category**	n *p*-Value (a)	1499	1499	0.187	1499	**0.002**
L0		1131 (75.5)	593 (52.4)	538 (47.6)	484 (42.8)	647 (57.2)
L1		368 (24.5)	178 (48.4)	190 (51.6)	192 (52.2)	176 (47.8)
**V-Category**	n *p*-Value (a)	1498	1498	1.000	1498	0.834
V0		1400 (93.5)	721 (51.5)	679 (48.5)	721 (51.5)	679 (48.5)
V1		98 (6.5)	50 (51.0)	48 (49.0)	50 (51.0)	48 (49.0)
**Pn-Category**	n *p*-Value (a)	677	677	1.000	677	0.512
Pn0		638 (94.2)	338 (53.0)	300 (47.0)	281 (44.0)	357 (56.0)
Pn1		39 (5.8)	21 (53.8)	18 (46.2)	15 (38.5)	24 (61.5)
**Grading**	n *p*-Value (a)	1476	1476	0.738	1176	**0.002**
Low grade (G1/G2)		1202 (81.4)	616 (51.2)	586 (48.8)	521 (43.3)	681 (56.7)
High grade (G3/G4)		274 (18.6)	144 (52.6)	130 (47.4)	147 (53.6)	127 (46.4)
**R-Status**	n *p*-Value (a)	1458	1458	0.097	1458	0.408
R0		1421 (97.5)	739 (52.0)	682 (48.0)	640 (45.0)	781 (55.0)
R1/R2		37 (2.5)	14 (37.8)	23 (62.2)	14 (37.8)	23 (62.2)
**Mismatch repair protein (MMR) status**	n p-Value (a)	487	487	**0.002**	487	0.016*
MMR proficient		392 (80.5)	193 (49.2)	199 (50.8)	171 (43.6)	221 (56.4)
MMR deficient		95 (19.5)	64 (67.4)	31 (32.6)	55 (57.9)	40 (42.1)
**KRAS mutation status**	n p-Value (a)	26	26	0.453	26	0.683
KRAS wild-type		14 (53.8)	7 (50.0)	7 (50.0)	4 (28.6)	10 (71.4)
KRAS mutation		12 (46.2)	8 (66.7)	4 (33.3)	5 (41.7)	7 (58.3)
**Overall Survival [Months]**	*p*-Value (c)		1495	0.648	1495	0.051
Total / Events / Censored		1495/737/758	770/378/392	725/359/366	672/352/320	823/385/438
Median Survival		92.4	95.5	85.8	70.4	119.6
95% C.I.		114.8–125.2	n.c.	n.c.	48.2–92.7 (+ − 11.4)	n.c.
**Tumour Specific Survival [Months]**	*p*-Value (c)		1481	0.193	1481	0.093
Total / Events / Censored		1481/501/980	763/246/517	718/255/463	665/240/425	816/261/555
Median Survival		n.c.	n.c.	n.c.	n.c.	n.c.
95% C.I.		141.6–152.1	n.c.	n.c.	n.c.	n.c.

### Immunohistochemistry

We evaluated 4497 CRC samples from 1499 patients for the expression of IGF1R in tumor cells.

A weak membranous immunostaining of tumor cells (mCC-IGF1R 1+) was observed in 910 (60.7%) cases and a strong membranous staining (mCC-IGF1R 2+) in 230 (15.3%) cases. Cells without membranous immunoreactivity (mCC-IGF1R 0) were seen in 1472 (98.2%) of all cases.

The percentage of immunostained tumor cells ranged from 0 to 100% and the combination of immunostaining categories varied in each individual sample. In 588 (39.2%) of all samples no membranous IGF1R expression was observed. In 2 cases (0.1%) all tumor cells (100%) showed a mCC-IGF1R 1+ staining and in 2 cases (0.1%) 90% of all tumor cells depicted a mCC-IGF1R 2+ staining. All other CRC samples showed various combinations of each staining intensity. The median HScore for mCC-IGF1R was 10 (range 0–190). The study population was dichotomized into mCC-IGF1R-low (HScore ≤10) and mCC-IGF1R-high (> 10). 771 (51.4%) of all CRCs were mCC-IGF1R-low and 728 (48.6%) were mCC-IGF1R-high.

A strong cytoplasmic immunostaining of tumor cells (cCC-IGF1R 2+) was observed in 202 (13.5%) and a weak cytoplasmic immunostaining (cCC-IGF1R 1+) in 1280 (85.4%) CRCs. Tumor cells lacking cytoplasmic IGF1R immunostaining (cCC-IGF1R 0) were found in 927 (61.8%) cases. The three cytoplasmic immunostaining categories covered different percentage areas within each CRC sample, ranging from 0% up to 100% respectively. In 219 (14.6%) CRC samples, no cytoplasmic immunostaining was detectable in any of the tumor cells. In 402 (26.8%) cases all tumor cells showed a weak (cCC-IGF1R 1+) immunostaining and in 4 (0.3%) cases 90% of all tumor cells within a given sample showed a strong cytoplasmic IGF1R expression (cCC-IGF1R 2+).The median HScore for cCC-IGF1R was 90 (range 0–190). The study population was dichotomized into cCC-IGF1R-low (HScore < 90) and cCC-IGF1R-high (≥90). 676 (45.1%) CRCs were cCC-IGF1R-low and 823 (54.9%) CRCs were cCC-IGF1R-high.

### Correlation of IGF1R – expression with clinicopathological data

To evaluate the biological relevance of IGF1R expression in CRC, we correlated cCC-IGF1R and mCC-IGF1R with clinicopathological patient characteristics (Table 1), respectively. Tumors of CRC patients with cCC-IGF1R-high were significantly more frequently of a left-sided origin and of a lower (G1/G2) tumor grade. cCC-IGF1R-high was significantly associated with a lower T category in CRC. cCC-IGF1R-high was significantly more frequent in CRCs without lymph vessel invasion.

mCC-IGF1R-high was significantly associated with the MMR proficient phenotype as well as a left-sided location of the CRC. With respect to membranous IGF1R expression, no other associations were found.

The associations between cCC-IGF1R-high and a MMR proficient phenotype, a lower UICC stage and a diminished nodal spread lost their significance according to the Siemes (Benjamini-Hochberg) procedure for multiple testing.

### Survival analysis

**Fig. 2:**
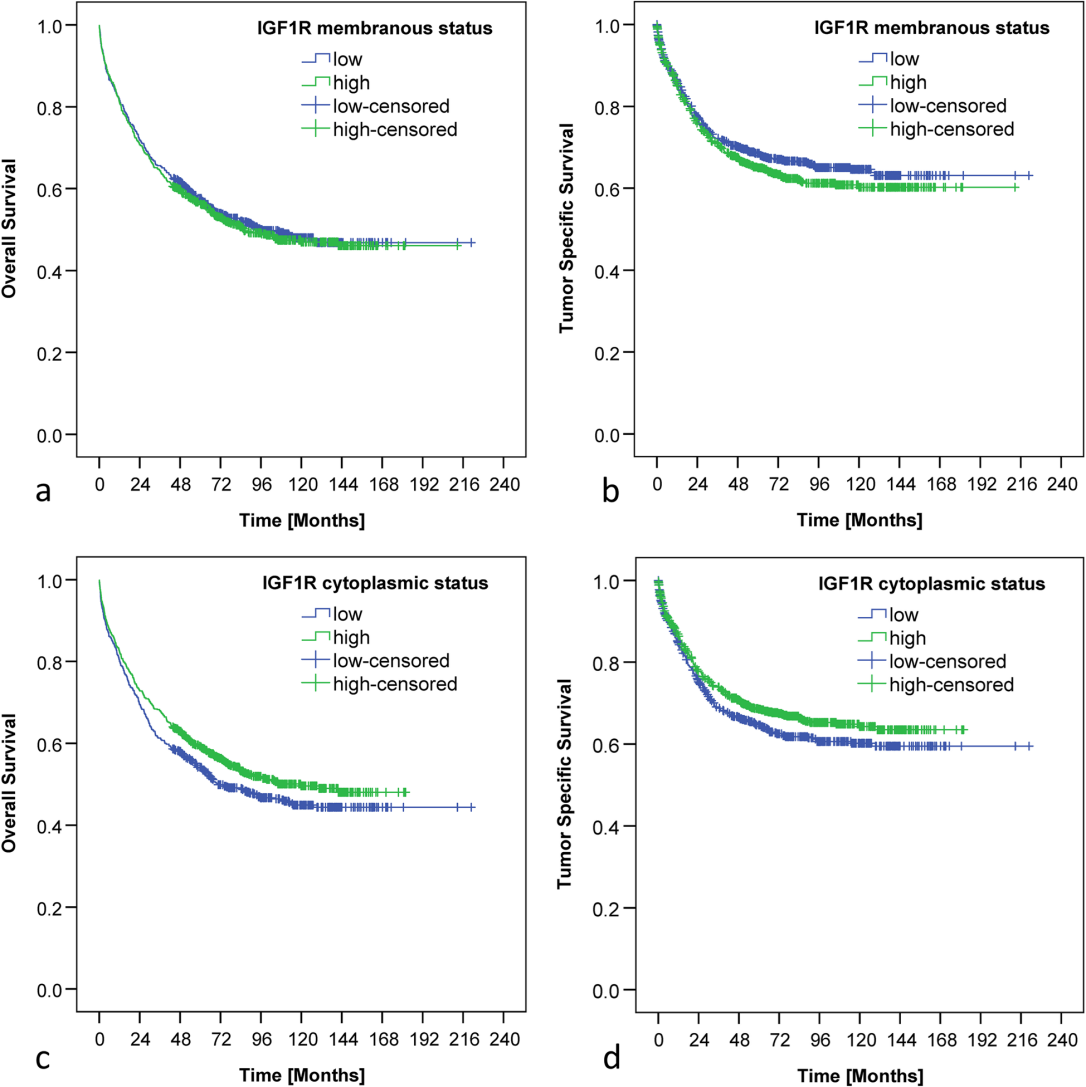
Kaplan-Meier curves. Kaplan-Meier curves demonstrating correlations between membranous IGF1 receptor expression in tumor cells and overall (**a**; *p* = 0.648) as well as tumor specific survival (**b**; *p* = 0.193). Kaplan-Meier curves demonstrating correlations between cytoplasmic IGF1 receptor expression in tumor cells and overall (**c**; *p* = 0.051) as well as tumor specific survival (**d**; *p* = 0.093). Numbers at risk are provided below each Kaplan-Meier curve

The mean overall survival (OS) of the whole CRC study population was 119.9 months and the mean tumor-specific survival (TSS) was 146.8 months. Prognosis was significantly associated with gender, age, T-, N-, M-category, UICC-stage, L-, V-, Pn-, R- category and grading (data not shown). CRC patients with cCC-IGF1R-high showed a strong tendency for longer overall survival, which missed statistical significance (*p* = 0.051). No significant associations between IGF1R expression and survival were found (Fig. 2).

**Fig. 3:**
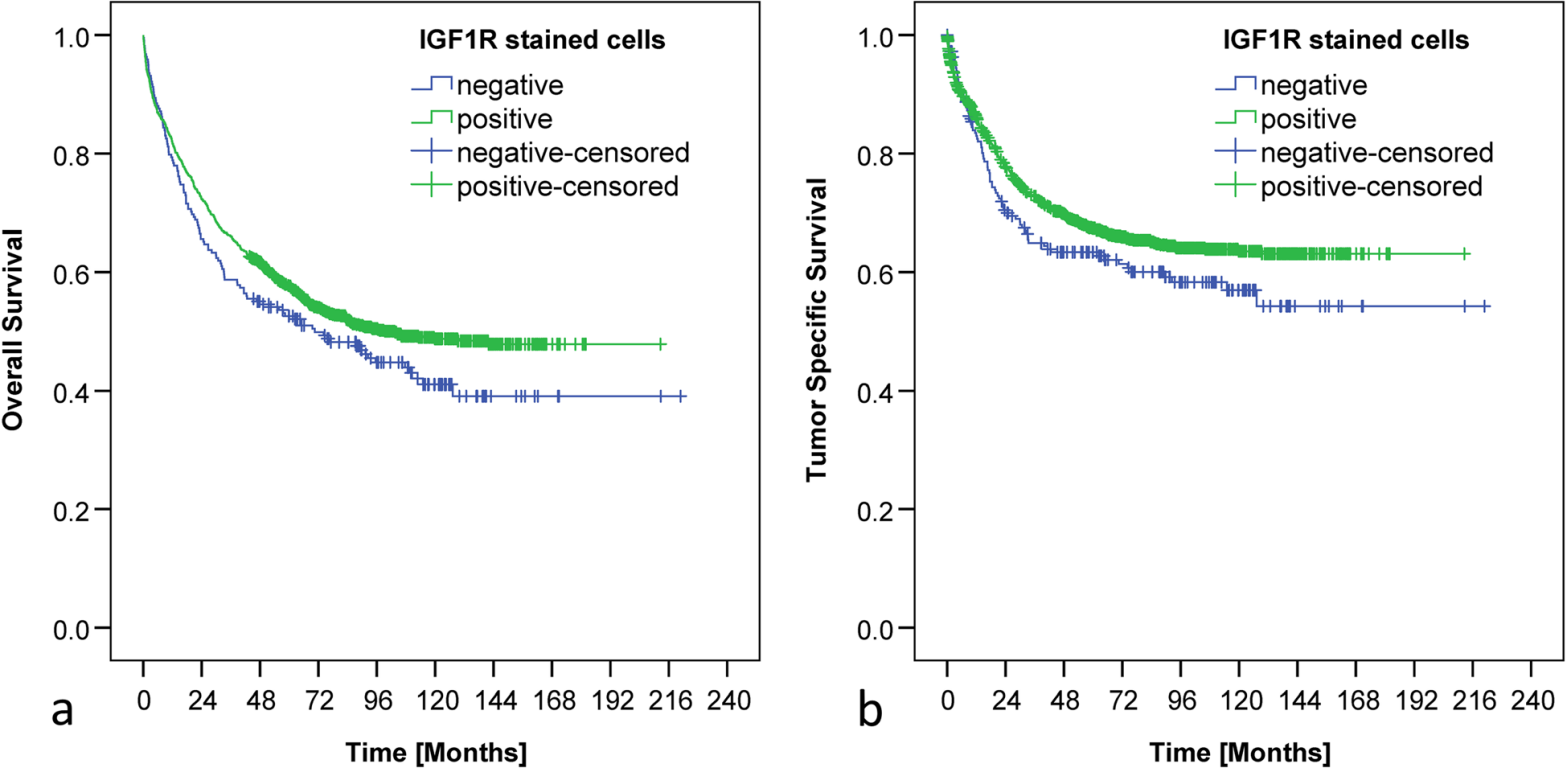
Kaplan-Meier curves based on the percentage of stained cells. Kaplan-Meier curves demonstrating correlations between IGF1 receptor expression in tumor cells and overall (**a**; *p* = 0.076) and tumor-specific (**b**; p = 0.076) survival based on the evaluation of the percentage of stained cells. CRCs with < 10% IGF1R positive tumor cells were declared as IGF1R negative and CRCs bearing ≥10% IGF1R positive tumor cells were classified as IGF1R positive. Neither the staining intensity nor the compartmental localization of the IGF1R were incorporated. Numbers at risk are provided below each Kaplan-Meier curve

In order to achieve an improved comparability with the results of other study groups [1, 6], we correlated IGF1R expression with survival by only evaluating the percentage of positively stained tumor cells. In this sense, CRC samples with cytoplasmic or membranous IGF1R expression in ≥10% of all tumor cells were classified as IGF1R positive. CRC samples which exhibited cytoplasmic as well as membranous IGF1R expression in less than 10% of all tumor cells were regarded as IGF1R negative. IGF1R positive CRCs tended to show an improved overall as well as tumor-specific survival, which missed significance (*p* = 0.076 and p = 0.076 respectively; Fig. 3).

### Correlation of IGF1R-expression with insulin receptor expression

IR expression data was available for 1457 out of 1499 CRC patients examined in the present study. The comparative analysis of IGF1R- and IR-expression therefore enclosed 1457 patients. cCC-IGF1R-high correlated significantly with IR expression in tumor cells (*p* < 0.001) and tumor vessels (*p* < 0.001) (Table 2).

**Table 2:** Correlation between the expression of the insulin-like growth factor receptor 1 (IGF1R) and the insulin receptor (IR) in tumor cells

	IR expression in tumor cells		IR expression in tumor vessels		
	negative	positive	low	high	
**Cytoplasmic IGF1R expression**	n (%)	n (%)	n (%)	n (%)	***p***-Value
** low (HScore < 90)**	217 (33.0)	440 (67.0)	287 (43.7)	370 (56.3)	< 0.001
** high (HScore ≥ 90)**	188 (23.5)	613 (76.5)	230 (28.7)	570 (71.3)	
**Membranous IGF1R expression**					
**low (HScore ≤ 10)**	224 (29.9)	526 (70.1)	319 (42.5)	431 (57.5)	< 0.001
**high (HScore > 10)**	181 (25.6)	527 (74.4)	198 (28.0)	509 (72.0)	

Membranous IGF1R-expression in tumor cells correlated significantly with IR expression in tumor vessels (*p* < 0.001) but not with IR status in tumor cells (*p* = 0.07). However, cytoplasmic IGF1R-expression correlated significantly with the IR status in tumor cells (*p* < 0.001) (Table 2), albeit.

## Discussion

On the basis of a large study population, our investigative cohort analysis of IGF1R expression in CRC leads to new results contrasting former studies.

Up to now, the common belief was that the IGF1R promotes CRC progression and is associated with worse survival. Takahari et al. had associated IGF1R expression with shorter survival in a cohort consisting of 91 CRC patients [2]. Shiratsuchi et al. had studied a cohort of 210 CRC patients and reported that IGF1R expression was more frequently seen in tumors of larger size [6]. In experimental CRC models, IGF1R inhibition exhibited antitumor effects in combination with chemotherapy [14]. Therefore IGF1R inhibition had been pursued in several clinical trials. Nevertheless, clinical trials failed to show efficacy of IGF1R inhibiting antibodies in CRC [7–9]. Cohn et al. tested the combination of the IGF1R-blocking antibody Ganitumab with a FOLFIRI chemotherapy regimen [8] and found no benefit of the additional IGF1R-inhibition.

In our study, we wanted to scrutinize the IGF1R’s role in CRC pathophysiology more elaborately by basing our investigation upon a large study population. We knew that only a large study population could prevent a potential type II error, which could have misled former investigators.

In our analysis of 1499 CRCs, we found no significant correlation of IGF1R expression with survival. We even observed a tendency for prolonged overall survival in CRC patients with high cytoplasmic IGF1R expression. Our survival data appear to be consistent with our associations between IGF1R expression and clinicopathological parameters: IGF1R expression was associated with more differentiated tumors, less lymphatic vessel invasion and a lower tumor size at diagnosis. We therefore postulate that the IGF1R has been suspected wrongfully to promote worse survival in CRC.

Our results oppose not only former, but even a more recent study published by Han et al. in 2016 involving 121 CRC patients [1]. Han et al. described an association between IGF1R expression and worse survival in CRC and correlated IGF1R expression with higher tumor stages, poor differentiation and lymphatic metastasis.

Although we think that our study is based on a broad foundation, we have to consider potential confounders: Different evaluation schemes may explain the discrepant results. Different from Han et al. and other groups we used the HScore for the assessment of immunostaining and we distinguished between cytoplasmic and membranous IGF1R expression: The HScore aimed to improve stratification between tumor cells with low and high IGF1R expression, as we observed heterogeneity in IGF1R expression. Low IGF1R expressing tumor cells with a faint, but evident, immunostaining were not to shift the balance to the same extent as unambiguously high IGF1R expressing cells. The distinction between membranous and cytoplasmic IGF1R expression served to acknowledge IGF1R’s biological characteristics even furthermore, as we appreciated that a cytoplasmic localization of the IGF1R reflects a state of activation [15]. Our evaluation scheme therefore represents a further development beyond the black and white scheme, which we used for the evaluation of tumor cells in our former study about IR expression in CRC [4]. We are aware that the different evaluation schemes limit comparability between the former IR and the present IGF1R study. We are optimistic that the large sample size of our studies and the fact that the same TMA cores were used for both studies, should level potential effects arising from different approaches.

Other groups have not yet employed such an approach for the evaluation of IGF1R expression in CRC: The study conducted by Han et al. only evaluated the percentage of IGF1R expressing tumor cells, but not staining intensity [1]. Furthermore, membranous and cytoplasmic staining were not distinguished. Shiratsuchi et al. [6], who associated IGF1R expression with larger tumor sizes, employed a cut-off value of 10% IGF1R immunopositive tumor cells irrespective of the staining intensity without providing a rational for the cut-off value. In order to rule out different evaluation schemes as the reason for discrepant results, we performed a new survival analysis based on the evaluation of the percentage of stained cells only. Our new survival analysis, though now being readily comparable, still opposes the results of the aforementioned studies. Up to our knowledge, only a single study pointed in the same direction, when Nakamura et al. described a correlation between high IGF1R expression and a decreased risk of recurrence in Dukes C CRC [5]. Unfortunately, the study population was small (*n* = 161 CRC patients) and no correlations were found with any other clinicopathological patient characteristics [5].

Interestingly, our study revealed a previously unknown association between IGF1R expression and the MMR expression status in CRC. It might seem surprising to observe an association with IGF1R expression, as the MMR proficient CRC phenotype had been associated with worse survival by some study groups [16]. It has to be kept in mind that other study groups have found no association with survival such as Gkekas et al. [17] and that even our own data only shows a tendency - without reaching significance - for an improved survival of CRC patients with dMMR [4]. The MMR proficient CRC phenotype and IGF1R expressing CRCs basically share two key characteristics, which might explain their association: both reflect a higher degree of differentiation and both are associated with left-sidedness.

Up to now, IGF1R directed antibody therapy of tumor entities other than CRC showed no promising results in clinical trials. Several hypotheses exist, which try to explain the cause for the trials’ failures. One major hypothesis stated that IR expression might be upregulated upon IGF1R inhibition [18] - the IR might compensate IGF1R blockage, as IGF1R ligands also bind the IR, in particular the IR’s mitogenic isoform A [18]. We proved in a former study that IR isoform A is upregulated in CRC [4]. We now show for the first time that IGF1R- and IR-expression are associated in CRC. It is therefore hypothetically conceivable that IGF1R-inhibition might be compensated by IR signaling in CRC in the context of the IR−/IGF1R-axis. Future studies have to evaluate if a therapeutic approach, which combines the inhibition of IGF1R and IR isoform A in CRC, might prove to be more successful. As the IGF1R and IR were predominantly expressed in lower tumor stages, this therapeutic approach would be limited to early CRCs. Then again, as no association between IGF1R expression and survival was found in the present study and IR expressing CRCs were found to have an even improved survival [4], it remains questionable, if even an IGF1R / IR-A-targeted dual therapy is suitable for this tumor type. The IGF1R seems to be beyond suspicion in CRC after all.

## Conclusions

IGF1R expression is frequent and biologically relevant in CRC and was associated with tumor localization, an MMR proficient CRC phenotype, more differentiated tumors, less lymphatic vessel invasion and a lower tumor size at diagnosis. We found no association between IGF1R expression and survival in CRC, although a strong tendency for longer overall survival was seen for cytoplasmic IGF1R expression. We conclude that IGF1R expression has been suspected wrongfully to promote worse survival in CRC.

## Data Availability

The datasets generated during and/or analyzed during the current study are included in this published article and are otherwise available from the corresponding author on reasonable request.

## Abbreviations

IGF1R: insulin-like growth factor 1 receptor
CRC: colorectal cancer
IR: insulin receptor
TMA: tissue microarrays
TBS: Tris-buffered saline
HScore: HistoScore
OS: Overall survival
TSS: Tumor-specific survival
cCC-IGF1R: Cytoplasmic IGF1R immunostaining of cancer cells
mCC-IGF1R: Membranous IGF1R immunostaining of cancer cells
MMR: Mismatch repair protein
